# Isolated single atom cobalt in Bi_3_O_4_Br atomic layers to trigger efficient CO_2_ photoreduction

**DOI:** 10.1038/s41467-019-10392-w

**Published:** 2019-06-28

**Authors:** Jun Di, Chao Chen, Shi-Ze Yang, Shuangming Chen, Meilin Duan, Jun Xiong, Chao Zhu, Ran Long, Wei Hao, Zhen Chi, Hailong Chen, Yu-Xiang Weng, Jiexiang Xia, Li Song, Shuzhou Li, Huaming Li, Zheng Liu

**Affiliations:** 1 Center for Programmable Materials, School of Materials Science & Engineering, Nanyang Technological University, Singapore, 639798 Singapore; 2 School of Chemistry and Chemical Engineering, Institute for Energy Research, Jiangsu University, 301 Xuefu Road, Zhenjiang, 212013 China; 3 National Synchrotron Radiation Laboratory, CAS Center for Excellence in Nanoscience, School of Chemistry and Materials Science, University of Science and Technology of China, Hefei, Anhui 230029 China; 4 Materials Science and Technology Division, Oak Ridge National Laboratory, Oak Ridge, 37830 USA; 5 Laboratory of Soft Matter Physics, Institute of Physics, Chinese Academy of Sciences, Beijing, 100190 China

**Keywords:** Inorganic chemistry, Solar fuels, Photocatalysis, Two-dimensional materials

## Abstract

The design of efficient and stable photocatalysts for robust CO_2_ reduction without sacrifice reagent or extra photosensitizer is still challenging. Herein, a single-atom catalyst of isolated single atom cobalt incorporated into Bi_3_O_4_Br atomic layers is successfully prepared. The cobalt single atoms in the Bi_3_O_4_Br favors the charge transition, carrier separation, CO_2_ adsorption and activation. It can lower the CO_2_ activation energy barrier through stabilizing the COOH* intermediates and tune the rate-limiting step from the formation of adsorbed intermediate COOH* to be CO* desorption. Taking advantage of cobalt single atoms and two-dimensional ultrathin Bi_3_O_4_Br atomic layers, the optimized catalyst can perform light-driven CO_2_ reduction with a selective CO formation rate of 107.1 µmol g^−1^ h^−1^, roughly 4 and 32 times higher than that of atomic layer Bi_3_O_4_Br and bulk Bi_3_O_4_Br, respectively.

## Abstract (web summary)

While the conversion of CO_2_ to high-value products provides a promising means to remove and utilize atmospheric carbon, few materials can do so without wasteful, sacrificial reagents. Here, authors prepare single-atom Co on Bi_3_O_4_Br nanosheets as CO_2_ reduction catalysts using water and light.

## Introduction

Photocatalytic CO_2_ reduction with water as reaction medium to yield value-added carbon products has been regarded as an appealing approach to remit the energy issue and manage the global carbon balance simultaneously^1–5^. Despite a good deal of impressive photocatalysts have been developed for CO_2_ reduction, most of them are still subjected to low photocatalytic activity, poor product selectivity or the requirement of sacrificial agent, which greatly limit the possible practical applications. Hence, it is desirable to design robust catalysts with high reduction efficiency, high selectivity without the utilization of sacrifice reagent.

Recently, ultrathin two-dimensional (2D) materials with suitable energy band structure have been demonstrated distinct advantages as one class of emerging photocatalysts^6–10^. The atomic thickness with concomitant huge specific surface area allows better absorption of ultraviolet-visible light. The ultrathin configuration can significantly decrease the bulk recombination possibility of charge carriers owning to the shortened diffusion distance from inside to surface. Moreover, the high ratio of coordination-unsaturated surface atoms to overall atoms can afford more sites facilitating the interfacial reactions^11^. Especially, the single species of surface atoms is conducive to the production of highly selective CO_2_ reduction products^12–14^. Up to now, several types of ultrathin nanosheets have been developed and employed for CO_2_ photoreduction, such as WO_3_ layers^12^, ultrathin ZnAl LDH^13^, single-unit-cell o-BiVO_4_^14^, Bi_2_WO_6_ layers^15^, and one-unit-cell ZnIn_2_S_4_^16^. However, the high surface charge recombination rate and lack of active sites limits the sufficient utilization of charge carriers to trigger the photoreduction process. In order to further improve the CO_2_ reduction efficiency, incorporating isolated single atoms into 2D ultrathin nanosheets may be an appealing strategy. With maximum atom-utilization efficiency and unique properties, single-atom catalysts (SAC) display enormous potential in different catalytic applications^17,18^. Considering the cobalt (Co) SAC show outstanding performance toward electrocatalytic CO_2_ reduction due to the unique electronic structure, it is desirable to introduce Co single atoms as active sites to build Co SA/2D materials and employed for CO_2_ photoreduction^19,20^. Moreover, this allocation affords a favorable platform to in-depth insight the structure–property relationship between doped single atoms and the corresponding photocatalytic activity. Herein, taking the ultrathin Bi_3_O_4_Br nanosheets as a prototype, isolated single-atom Co is incorporated into Bi_3_O_4_Br atomic layers to build a Co–Bi_3_O_4_Br catalyst and employed for CO_2_ photoreduction.

## Results

### Characterizations of Co–Bi_3_O_4_Br nanosheets

**Fig. 1:**
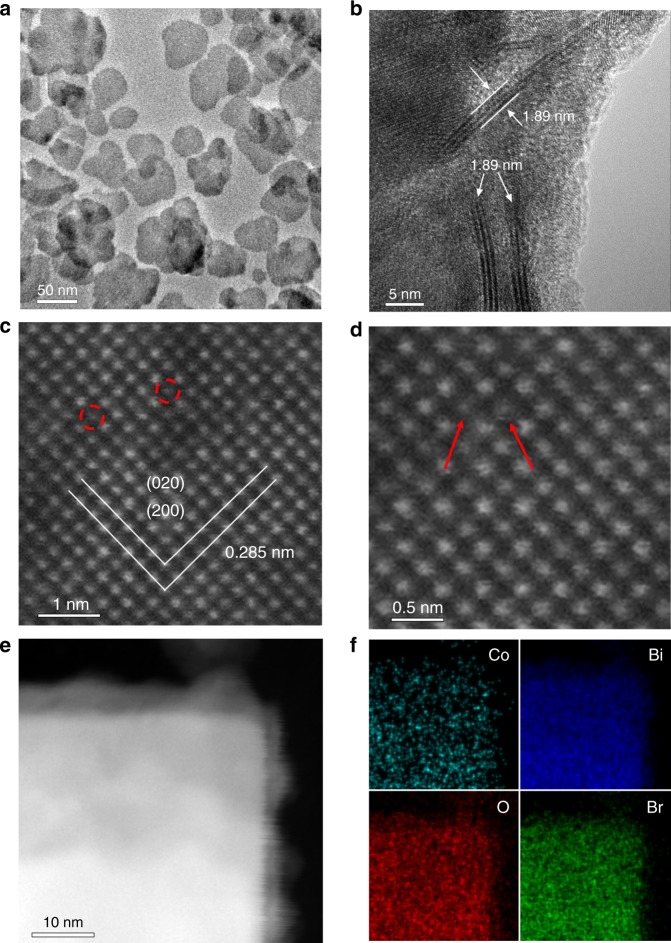
Surface morphology of Co–Bi_3_O_4_Br-1. **a**, **b** TEM and **c**, **d** atomic resolution HAADF-STEM images of Co–Bi_3_O_4_Br-1, **e**, **f** STEM image and EDS mapping images of Co, Bi, O, and Br

X-ray diffraction (XRD) and Raman spectra suggest the successful preparation of orthorhombic Bi_3_O_4_Br and the Co incorporation did not destroy the crystal structure (Supplementary Fig. 1). The transmission electron microscopy (TEM) image in Supplementary Fig. 2a–c depicts a sheet-like morphology of Bi_3_O_4_Br with the thickness of 1.89 nm, corresponding to unit cell (*c* parameter) thickness of Bi_3_O_4_Br. The observed lattice spacing of 0.285 nm is corresponded to the (020) or (200) crystal plane spacing of Bi_3_O_4_Br, revealing the (002) facet exposure (Supplementary Fig. 2d). The TEM and atomic force microscope images of Co–Bi_3_O_4_Br-1 show the similar morphology and thickness with pure Bi_3_O_4_Br (Fig. 1a, b, Supplementary Fig. 3). To disclose the fine structure and distribution of Co, atomic resolution high-angle annular dark-field scanning TEM (HAADF-STEM) is performed. The isolated dark dots Fig. 1c, d can be unambiguously ascribed to individual Co atoms according to the Z-contrast difference between the light Co and the heavier Bi atoms^21^. The isolated Co atoms are substituting Bi atoms in the crystal lattice. In addition, elemental mapping with subnanometer resolution (Fig. 1e, f) shows a uniform, uncorrelated spatial distribution of Co with a content of 0.8 wt%.

**Fig. 2:**
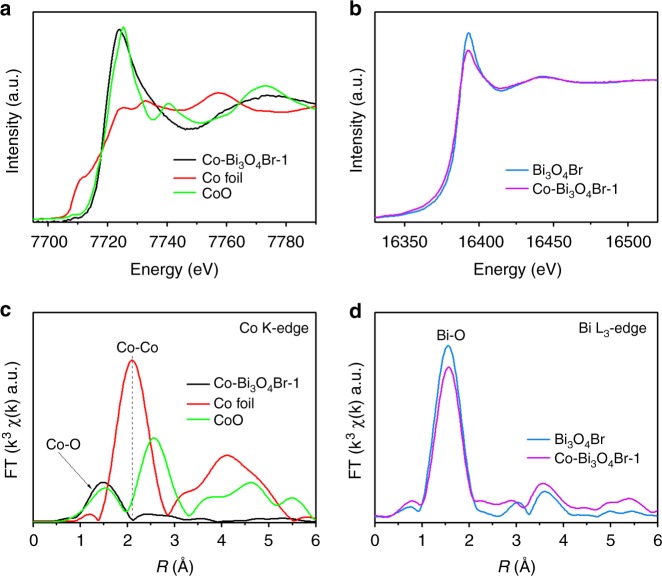
Synchrotron radiation XAFS measurements. **a** Co K-edge XANES spectra, **b** Bi L_3_-edge XANES spectra, EXAFS spectra of **c** Co K-edge and **d** Bi L_3_-edge

To further disclose the Co coordination environment, X-ray absorption near-edge structure and extended X-ray absorption fine structure (EXAFS) spectroscopy are performed. The difference in position and intensity of pre-edge peak at about 7711 eV signify that the Co atoms in the samples are of diverse environments (Fig. 2a)^22^. The Co K-edge absorption edge position of Co–Bi_3_O_4_Br-1 is located close to CoO rather than Co foil, suggesting single Co atom carries positive charge with the valence state is approach +2. The Bi L_3_-edge absorption edge position of Co–Bi_3_O_4_Br-1 shows slight difference with that of Bi_3_O_4_Br, suggesting distinct local atomic structure due to Co incorporation (Fig. 2b). View from the Fourier transformed (FT) k^3^-weighted EXAFS spectra (Fig. 2c), Co–Bi_3_O_4_Br-1 materials does not emerge the peak of Co–Co bond in reference to standard Co foil and CoO, revealing the absence of Co or CoO clusters/particles. These results demonstrate that the Co species are isolated single atoms. The main peak at 1.49 Å for Co–Bi_3_O_4_Br-1 is corresponding to the coordination with O atoms in Bi_3_O_4_Br lattice^22^. Beyond that, the main peak at around 1.5 Å in Bi L_3_-edge EXAFS spectra is assigned to the Bi–O bonds, in which the intensity of Co–Bi_3_O_4_Br-1 is weaker than that of Bi_3_O_4_Br (Fig. 2d)^23^. All these result qualitatively verify their distinct local atomic arrangement of Co–Bi_3_O_4_Br-1 relative to the Bi_3_O_4_Br, also certified by the peak shifting in X-ray photoelectron spectra (XPS, Supplementary Fig. 4) and decreased zeta potentials from 29.9 mV for Bi_3_O_4_Br to 18.4 mV for Co–Bi_3_O_4_Br-1 (Supplementary Fig. 5).

### Photocatalytic CO_2_ reduction performances

**Fig. 3:**
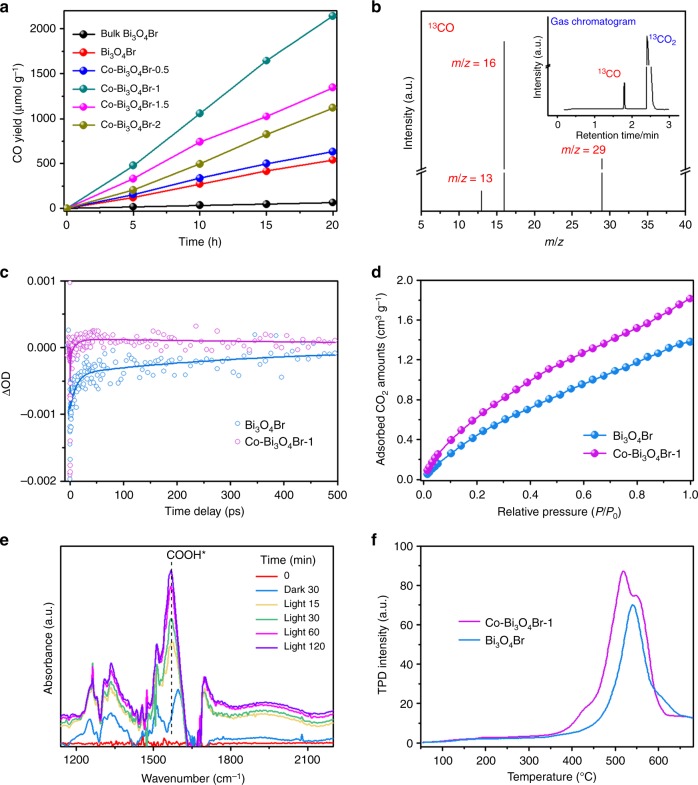
Evaluation of CO_2_ photoreduction performance and mechanism insight. **a** Photoreduction of CO_2_ into CO over Bi_3_O_4_Br and Co–Bi_3_O_4_Br materials, **b** mass spectra of ^13^CO (*m*/*z* = 29) produced over Co–Bi_3_O_4_Br-1 in photoreduction of ^13^CO_2_, **c** ultrafast TA spectra of Bi_3_O_4_Br and Co–Bi_3_O_4_Br, **d** CO_2_ adsorption isotherms of Bi_3_O_4_Br and Co–Bi_3_O_4_Br-1, **e** in situ FTIR spectra for the CO_2_ reduction process on the Co–Bi_3_O_4_Br-1, **f** CO TPD spectra of Bi_3_O_4_Br and Co–Bi_3_O_4_Br-1

The photocatalytic CO_2_ reduction performance of the samples is examined in neutral water under simulated solar light irradiation without any sacrificial reagents or photosensitizers. As shown in Fig. 3a, the Bi_3_O_4_Br atomic layer displays a CO formation rate of 27.0 µmol g^−1^ h^−1^, greatly higher than the 3.3 µmol g^−1^ h^−1^ for bulk Bi_3_O_4_Br (Supplementary Fig. 6), revealing the advantage of 2D ultrathin configuration. After the isolated single-atom Co is incorporated into Bi_3_O_4_Br atomic layers, the photocatalytic activity can be further improved. Among the diverse Co–Bi_3_O_4_Br materials, the Co–Bi_3_O_4_Br-1 displays the optimal performance. During a 20 h photocatalysis test, the total yield of CO can arrive 2142.1 µmol g^−1^, accompanied by a trace amount of methane (~3.28 µmol g^−1^). The average CO-generation rate of Co–Bi_3_O_4_Br-1 is up to 107.1 µmol g^−1^ h^−1^, roughly 4 and 32 times higher than that of Bi_3_O_4_Br atomic layer and bulk Bi_3_O_4_Br, respectively. This value is also higher than many ultrathin materials under the same testing conditions (Supplementary Fig. 7) and superior to many reported results (Supplementary Table 1). During the photoreduction process, Co–Bi_3_O_4_Br-1 sample can simultaneously achieve H_2_O oxidation into O_2_ with an average O_2_ evolution rates of about 56 µmol g^−1^ h^−1^ (Supplementary Fig. 8). The ratio of CO evolution rates to O_2_ evolution rates is 1.91, approach to the stoichiometric ratio of 2. Through tune the different usage amount of photocatalyst for CO_2_ reduction, the higher converted value of μmol g^−1^ h^−1^ can be achieved when less usage amount is employed (Supplementary Fig. 9). The control experiments in dark, under Ar condition or without catalyst did not show the evolution of CO, implying the CO is indeed produced by CO_2_ photoreduction. The ^13^CO_2_ isotopic labeling experiment is performed, in which the peak at *m*/*z* = 29 (^13^CO) can be observed, further affirming the formation of CO is indeed derived from the reduction of CO_2_ (Fig. 3b). Furthermore, the apparent quantum yields for Co–Bi_3_O_4_Br-1 are calculated to be approximately 0.91% and 0.36% at 380 and 400 nm, respectively. To determine the stability, the XRD, XPS valence-band spectra and TEM are employed for the used Co–Bi_3_O_4_Br-1 samples (Supplementary Fig. 10). No obvious variations of the crystal structure, electronic structure and morphology can be observed after the photoreaction, suggesting the favorable photostability.

### Insight of the increased photocatalytic activity

To elucidate the origin of the increased photocatalytic activity, three elementary processes in photocatalytic CO_2_ reduction namely light absorption, charge separation, and interfacial CO_2_ catalysis are taken into consideration^24^. After the incorporation of single-atom Co, the absorption in the visible light area can be improved due to the formed dopant energy levels of Co in the bandgap of Bi_3_O_4_Br (Supplementary Fig. 11a). The electrons in the valence band (VB) can be excited to the newly formed localized state of high-spin Co^2+^ (3d^7^). Moreover, the fully occupied electrons in t2 levels of Co^2+^ can be easily excited to the unoccupied e levels (d–d internal transitions), also contribute to the improved light absorption^25^. The increased density of states of Co–Bi_3_O_4_Br in the bandgap through density functional theory (DFT) calculation further confirm the easily transition of photogenerated electrons to the new energy levels (Supplementary Fig. 11c, d). The corresponding bandgap energy of Bi_3_O_4_Br and Co–Bi_3_O_4_Br-1 are calculated to be 2.29 and 2.21 eV, respectively (Supplementary Fig. 11b). The detailed energy-level positions of VB edges determined from XPS VB spectra are both 1.06 eV (Supplementary Fig. 11e). Thus, the conduction band (CB) potentials of Bi_3_O_4_Br and Co–Bi_3_O_4_Br-1 are determined to be −1.23 and −1.15 eV, respectively, satisfying the thermodynamic requirements for CO_2_ reduction to yield CO (Supplementary Fig. 11f).

To study the dynamic behaviors of photogenerated charge carriers in the prepared samples, ultrafast transient absorption (TA) spectra is employed. The biexponential fitting results are τ1 = 12 ps and τ2 = 400 ps for Bi_3_O_4_Br, while τ1 = 11 ps and τ2 = 1 ns for Co–Bi_3_O_4_Br-1 (Fig. 3c). Interestingly, the isolated single-atom Co nearly not alter τ1, but endows a ∼2.5-fold increase for τ2. The τ1 corresponded to electrons capture from CB into trap states within the bandgap, while the much slower decay component τ2 represents the recombination between the trapped electrons and the VB holes^16^. The engineered isolated single-atom Co can supply trap states to capture more photogenerated electrons, while the more long-lived, trapped electrons afford more opportunities for CO_2_ photoreduction. Moreover, time-resolved fluorescence emission decay spectra and surface photovoltage (SPV) spectroscopy are employed for further exploration (Supplementary Fig. 12a, b). The average fluorescence lifetime and SPV intensity of Co–Bi_3_O_4_Br-1 are much higher than that of Bi_3_O_4_Br atomic layers, respectively, further suggesting the increased charge separation efficiency in Co–Bi_3_O_4_Br. The isolated single-atom Co can work as charge separation center to trap the photogenerated electrons^26,27^, and thus increase the carrier utilization efficiency toward redox reactions, also certified by the transient photocurrent responses and electrochemical impedance spectroscopy (Supplementary Fig. 12c, d).

**Fig. 4:**
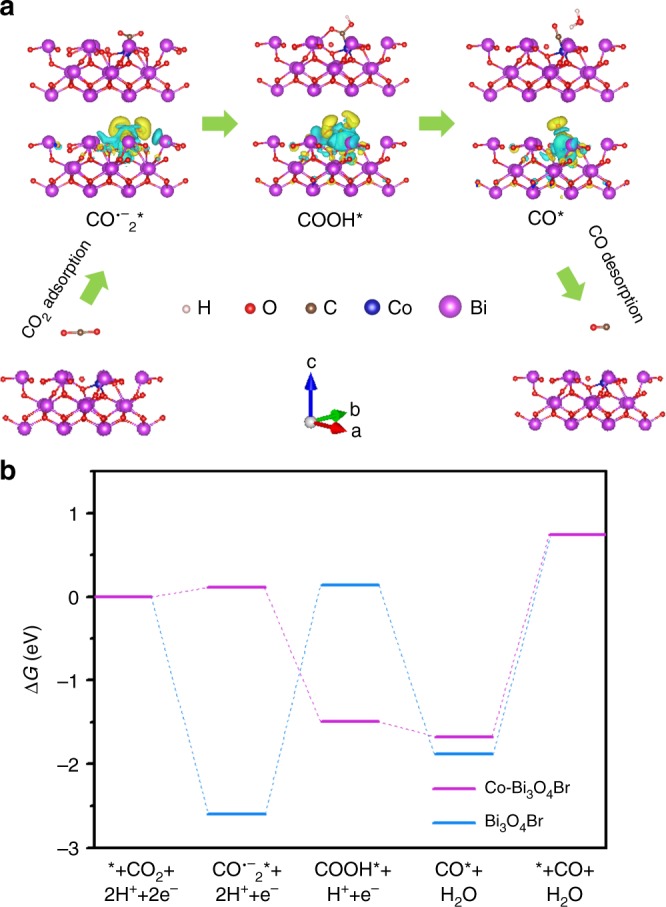
Theoretical study. **a** Schematic representation of CO_2_ photoreduction mechanism on the Co–Bi_3_O_4_Br, **b** free energy diagrams of CO_2_ photoreduction to CO for the Bi_3_O_4_Br and Co–Bi_3_O_4_Br

To dive deep into the interfacial catalysis of CO_2_ to yield CO, CO_2_ surface adsorption, activation, and CO desorption processes are explored. Firstly, the CO_2_ adsorption is generally considered as a prerequisite for CO_2_ photoreduction reaction. The isolated single-atom Co^2+^ by replacing Bi^3+^ enables the Co–Bi_3_O_4_Br atomic layers to be more negatively charged, which may in favor of CO_2_ adsorption on the surfaces^16^. It can be testified by the increased CO_2_ adsorption capacity of Co–Bi_3_O_4_Br-1 relative to Bi_3_O_4_Br, as illustrated in Fig. 3d. Contact-angle measurement demonstrates that hydrophilicity may be not the crucial factor to affect CO_2_ photoreduction reaction (Supplementary Fig. 13). Subsequently, the in situ Fourier transform infrared spectroscopy (FTIR) measurements are carried out to acquire in-depth understanding on the reaction intermediates (Fig. 3e). The peaks at 1256, 1337, and 1508 cm^−1^ can be assigned to CO_2_^−^, symmetric O–C–O stretches of b-CO_3_^2−^ and m-CO_3_^2^^−^ groups, respectively^28,29^. Notably the gradually increased peak at 1567 cm^−1^ is ascribed to COOH* intermediate, a type of critical intermediate during the formation of CO_2_ to CO^6,29^. The band at ~1600 cm^−1^ in the dark 30 min line is ascribed to the asymmetric O–C–O stretch of b-CO_3_^2−^ groups. With the prolonged light irradiation time, this band is obscured by the significantly increased COOH* band. Lastly, the CO desorption is also considered as an important factor to decide the entirely photocatalysis efficiency. As shown from CO temperature-programmed desorption (TPD), the Co–Bi_3_O_4_Br-1 exhibits lower onset desorption temperature and higher overall amount of detected CO, revealing the formed CO* molecules can liberate from the Co–Bi_3_O_4_Br-1 surface much easier (Fig. 3f)^28^. In addition, compared to the Bi_3_O_4_Br, a new lower-temperature desorption peak around 518 °C can be observed, implying the incorporated Co single atoms may favor the CO desorption, and this result is also certified by the calculated desorption free energy of CO (Fig. 4b).

## Discussion

According to the analysis, the possible CO_2_ reduction mechanism can be summarized as follows (Fig. 4a, Supplementary Fig. 14):

1 $${\mathrm{CO}}_2({\mathrm{g}}) \to {\mathrm{CO}}_2^ \ast$$

2 $${\mathrm{H}}_2{\mathrm{O}} \to {\mathrm{H}}^ + + {\mathrm{OH}}^ -$$

3 $${\mathrm{CO}}_{\mathrm{2}} \ast + \, {\mathrm{e}}^ - \to {\mathrm{CO}}_2^{ \cdot - } \ast$$

4 $${\mathrm{CO}}_2^{ \cdot - } \ast + \, {\mathrm{H}}^ + \to {\mathrm{COOH}}^ \ast$$

5 $${\mathrm{COOH}}^ \ast + {\mathrm{H}}^ + + {\mathrm{e}}^ - \to {\mathrm{CO}}^ \ast + {\mathrm{H}}_{\mathrm{2}}{\mathrm{O}}$$

6 $${\mathrm{CO}}^ \ast \to {\mathrm{CO}}$$

where “*” on behalf of the adsorption state at the materials surface.

The CO_2_ molecules are adsorbed on the surface of catalysts and the H_2_O molecules are dissociated into hydroxyl and hydrogen ions. The CO_2_* will be preferential combine with electron and hydrogen ion to form a carboxyl radical and then disintegrated into the adsorbed CO*^30^. Eventually, the adsorbed CO* will desorb from the catalyst surface to form gaseous CO.

To explore the reactivity nature and CO_2_ catalytic reduction cycle, DFT calculations are carried out (Fig. 4). Both the formation of COOH* and desorption of CO* for the Bi_3_O_4_Br are highly endergonic processes. The formation of adsorbed intermediate COOH* is found as the potential limiting step (Fig. 4b). However, the incorporated Co in Bi_3_O_4_Br can lower the CO_2_ activation energy barrier through stabilizing the COOH* intermediates and tuning the rate-limiting step to be CO* desorption.

In conclusion, isolated single-atom Co are incorporated into the Bi_3_O_4_Br atomic layers to generate photocatalysts with superior activity for CO_2_ reduction. Benefiting from the cooperation of ultrathin configuration and isolated single-atom Co, the Co–Bi_3_O_4_Br exhibits excellent photocatalytic activity toward CO_2_ reduction to high selective yield CO, with a high formation rate of 107.1 µmol g^−1^ h^−1^, roughly 4 and 32 times higher than that of Bi_3_O_4_Br atomic layer and bulk Bi_3_O_4_Br, respectively. The Co single atoms in the Bi_3_O_4_Br benefit the charge transition, charge-carrier separation kinetics, CO_2_ adsorption and activation. It can lower the CO_2_ activation energy barrier through stabilizing the COOH* intermediates and tunes the rate-limiting step from the formation of adsorbed intermediate COOH* to CO* desorption. Our findings shed light on the rational design of metal single atom incorporated atomic layer photocatalysts for robust solar-driven CO_2_ conversion performances.

## Methods

### Synthesis of Co–Bi_3_O_4_Br atomic layer

Totally, 0.5 mmol of Bi(NO_3_)_3_·5H_2_O, 0.0054 g cobalt(II) acetate tetrahydrate and 0.2 g polyvinyl pyrrolidone (PVP, K30) were dispersed into 15 mL mannitol solution (0.1 mol/L) to achieve solution A. Totally, 0.5 mmol NaBr dissolved into 3 mL mannitol solution (0.1 mol/L) to obtain solution B. Subsequently, solution B was added into solution A under stirring. After 30 min stirring, NaOH solution (2 M) was employed to tune the pH value to 11.5. Then suspension was sealed in a 25 mL teflon-lined stainless-steel autoclave and heated in oven at 160 °C for 24 h. After cooled down, the product was gathered, washed with deionized water and ethanol for several times, and dried. The calculated Co content relative to Bi_3_O_4_Br is 1 wt%, and the sample is named as Co–Bi_3_O_4_Br-1. Adjusting the Co content to 0, 0.5, 1.5 and 2 wt% to prepare pure Bi_3_O_4_Br, Co–Bi_3_O_4_Br-0.5, Co–Bi_3_O_4_Br-1.5, and Co–Bi_3_O_4_Br-2 samples.

### Synthesis of bulk Bi_3_O_4_Br

A total of 2 mmol Bi_2_O_3_ and 2 mmol BiOBr was sufficient mixed within 20 mL ethanol and stirred for 30 min. After drying, the powder was treated in muffle at 650 °C for 10 h with the ramping rate of 5 °C min^−1^ to achieve the product.

### Characterizations

The powder X-ray diffraction (XRD) were recorded on a Shimadzu XRD-6000 X-ray diffractometer with monochromatized Cu Kα radiation (*λ* = 0.15418 nm). The XPS spectra were collected by an ESCALab MKII X-ray photoelectron spectrometer and all binding energies were calibrated by using the contaminant carbon (C1s = 284.6 eV) as a reference. TEM images were collected on JEOL JEM-2100F. Aberration-corrected HAADF-STEM images were collected on a Nion Ultra STEM100 (USA) operated at 100 keV in Oak Ridge National Laboratory. Co K-edge and Bi L_3_-edge X-ray absorption fine structure measurements were performed at the beamline 14W1 in Shanghai Synchrotron Radiation Facility, China. UV–vis diffuse reflection spectra of the Bi_3_O_4_Br and Co–Bi_3_O_4_Br samples were recorded on a UV-2450 UV–vis spectrophotometer (Shimadzu, Japan). The photoluminescence (PL) spectra were conducted using a Varian Cary Eclipse spectrometer (USA). CO_2_ adsorption measurements were carried out through TriStar II 3flex gas adsorption analyzer (Micromeritics Instrument Corporation, USA). In situ FTIR were acquired using a Bruker vertex70. All electrochemical tests were performed on a CHI 660B electrochemical system (Chenhua Instruments) in conventional three-electrode cell with Pt as the counter electrode, and Ag/AgCl/sat. KCl electrode as the reference electrode. The ultrafast TA spectra were collected by using a femtosecond laser amplifier system (Spitfire Ace, Spectra Physics), which generates laser pulses with 800 nm central wavelength and ~35 fs pulse duration. The output beam was split into two beams. One was used to generate 400 nm pump light, and the other beam was focused into a sapphire plate, generating a broadband white light continuum probe beam. Both beams were focused onto the sample. After frequency resolved by a spectrograph, the excitation-induced transmission change for the probe light was collected by a home-built 46-channel synchronous digital lock-in amplifier. The isotope-labeled experiment was conducted using ^13^CO_2_ instead of ^12^CO_2_, and the products were analyzed through gas chromatography-mass spectrometry (7890A and 5975C, Agilent). CO TPD measurements were carried out on quantachrome autosorb-iQ-C chemisorption analyzer with a thermal conductivity detector.

### Calculation details

The first-principles simulations are conducted using the Vienna ab initio simulation package, the projector augmented wave potentials are used as pseudopotentials to describe the interactions between valence electrons and ions. The Perdew–Burke–Ernzerhof functional of generalized gradient approximation is used to describe the exchange-correlation of valence electrons. The lattice parameters of bulk Bi_3_O_4_Br is calculated first with the plane wave cutoff energy set as 500 eV and the k-point mesh set as 6 × 6 × 2. The convergence criteria are 10^−6^ eV in electric relaxation energy and 10^−4^ eV in ionic relaxation energy. The optimized lattice parameter for bulk Bi_3_O_4_Br was 5.78 Å × 5.80 Å × 19.03 Å. To calculate the Bi_3_O_4_Br layer slab, a 2 × 2 × 1 supercell is created and a 20 Å vacuum layer is added on top of the supercell to avoid inter-layer interactions. Thus, the lattice parameter of the layer slab model is 11.56 Å × 11.60 Å × 39.03 Å, with the plane wave cutoff energy set as 500 eV and the k-point mesh set as 3 × 3 × 1. For the case of cobalt substitution and CO_2_ molecule adsorption, all the parameters are remained same as that of Bi_3_O_4_Br layer slab, except that the convergence criteria for ionic relaxation is changed to 0.02 eV/Å instead.

### Photocatalytic CO_2_ reduction

The CO_2_ photoreduction performance evaluation of the Co–Bi_3_O_4_Br samples was carried out in a 500 mL Labsolar-6A closed gas system (Perfectlight, China). Totally, 30 mg samples was added into 50 mL water and well dispersed. The system was vacuum-treated and then pumped into high-purity CO_2_ with pressure of 0.08 MPa. Experiments were performed at 5 °C with a circulating water system to prevent thermal catalytic effects. The amount of CO and CH_4_ generated was determined using a gas chromatograph (Cotrun GC2002, FID) with a methanizer. A 300 W Xe lamp (Microsolar300, PerfectLight) was employed to provide light source. The apparent quantum efficiency is calculated according to the equation: apparent quantum efficiency = 100% × (number of generated CO × 2)/number of incident photons.

## Supplementary information

Supplementary Information

## Data Availability

The authors declare that the data supporting the findings of this study are available within the article and the Supplementary Information files.
