# Supplementary material for: Isolated single atom cobalt in Bi3O4Br atomic layers to trigger efficient CO2 photoreduction
Source: Nat Commun. 2019 Jun 28;10:2840. doi: 10.1038/s41467-019-10392-w (PMC6599015; doi:10.1038/s41467-019-10392-w)
Supplement: Supplementary file 1 — Supplementary Information [file 41467_2019_10392_MOESM1_ESM.pdf]

Isolated single atom cobalt in Bi_3O_4Br atomic layers to trigger efficient CO_2 photoreduction

Di et al.

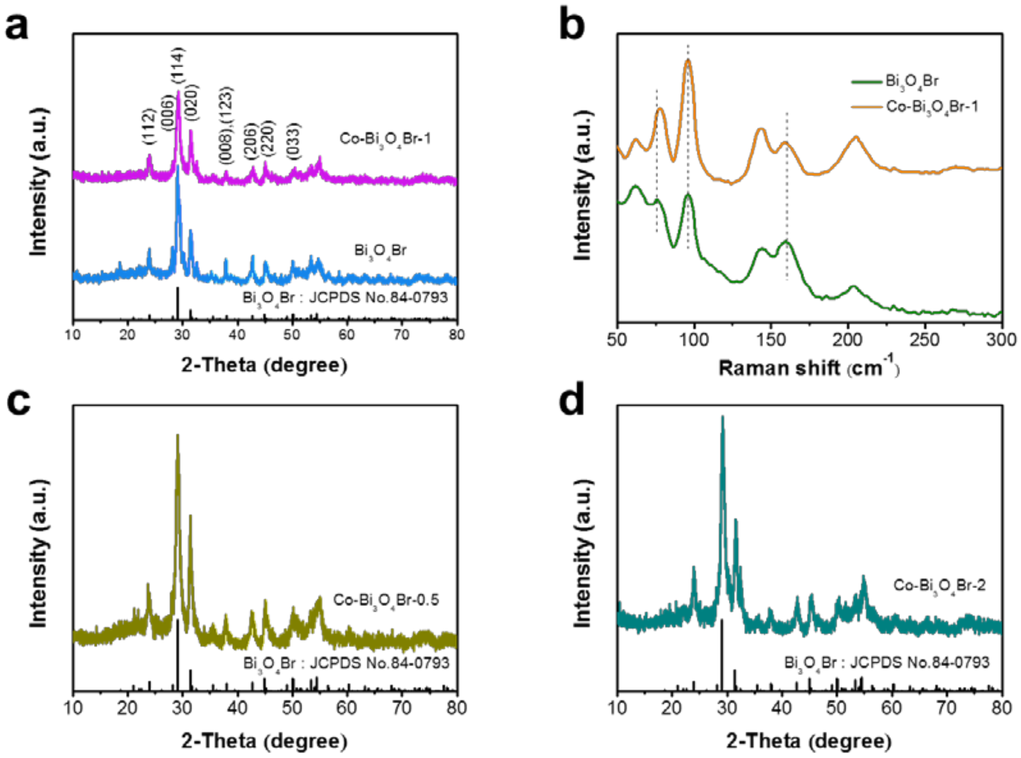

Supplementary Figure 1. Compositional characterization. (a) XRD pattern, (b) Raman spectrum of Bi_3O_4Br and Co-Bi_3O_4Br-1, (c) XRD pattern of Co-Bi_3O_4Br-0.5, (d) XRD pattern of Co-Bi_3O_4Br-2.

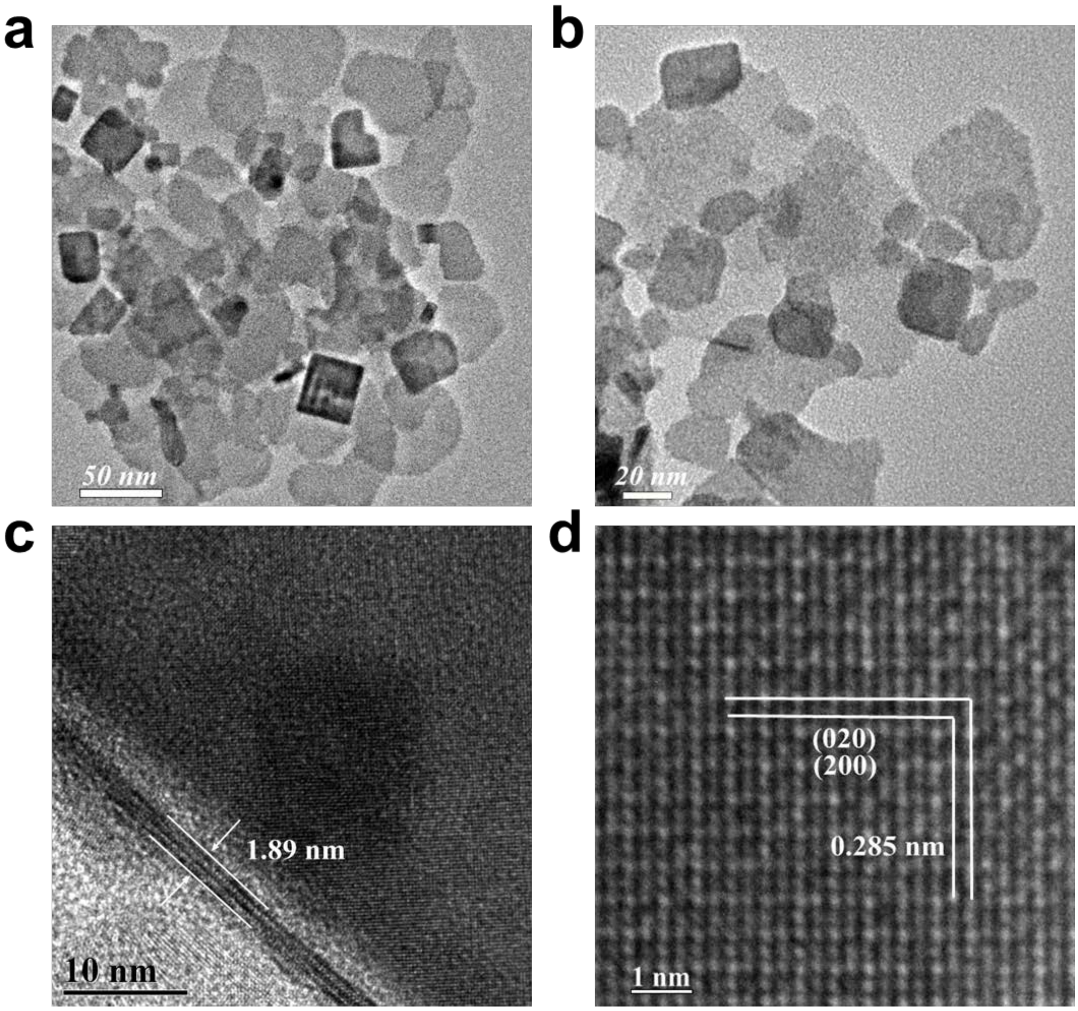

Supplementary Figure 2 TEM analysis. (a, b) TEM images and (c, d) HRTEM images of Bi_3O_4Br atomic layer.

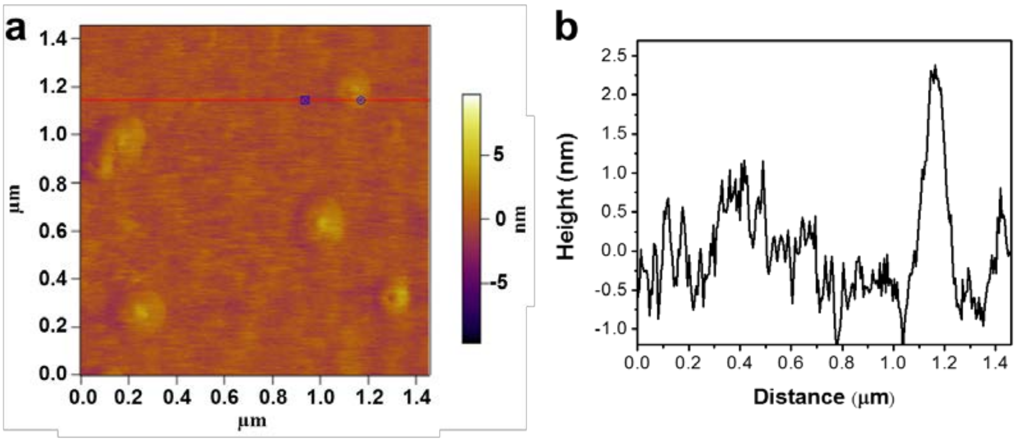

Supplementary Figure 3. AFM analysis. (a) AFM image, and (b) the corresponding height profiles of Co-Bi_3O_4Br-1 materials.

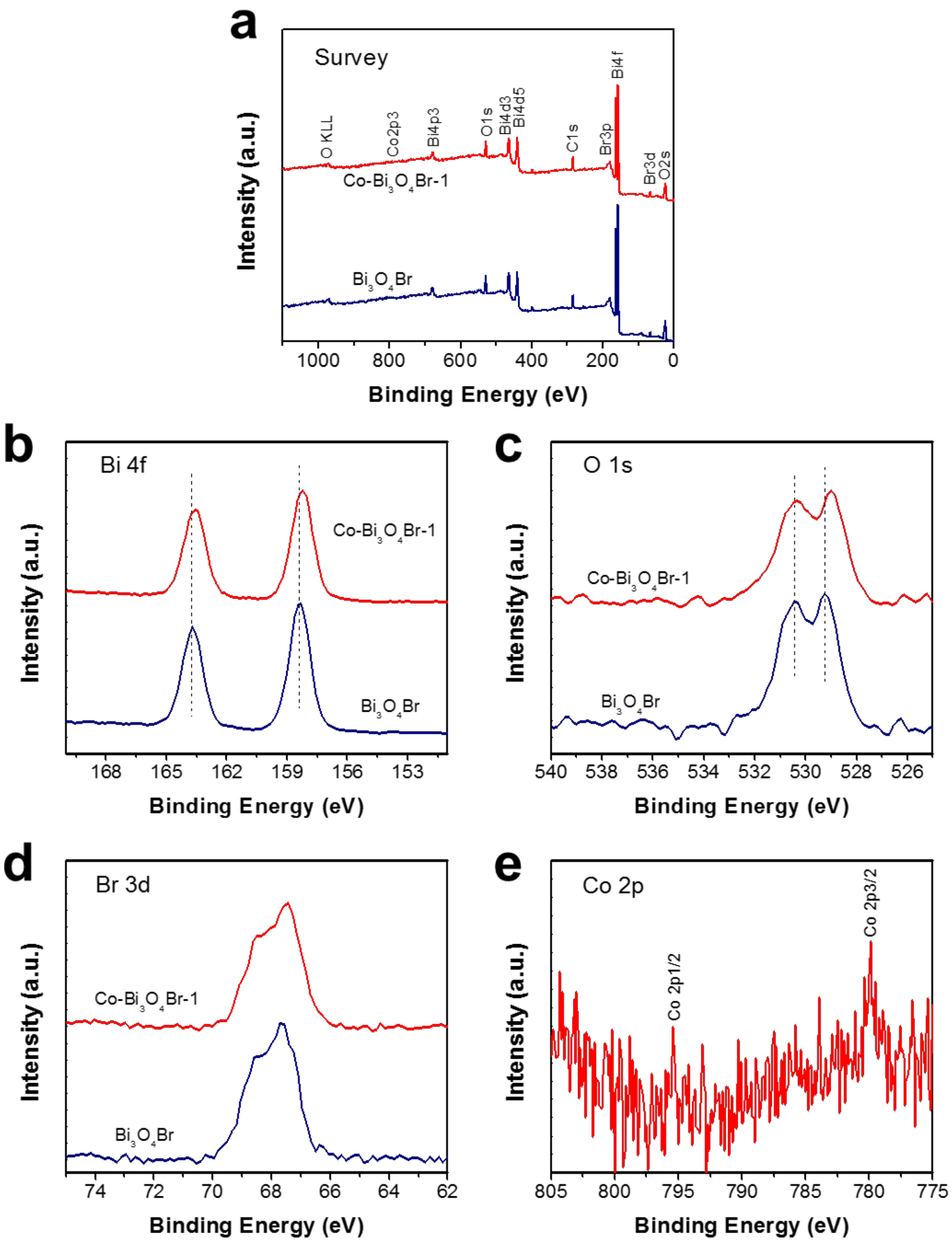

Supplementary Figure 4. XPS analysis. X-ray photoelectron spectra: (a) full scan, (b) Bi 4f, (c) O 1s, (d) Br 3d, and (e) Co 2p regions of Bi_3O_4Br and Co-Bi_3O_4Br-1 materials.

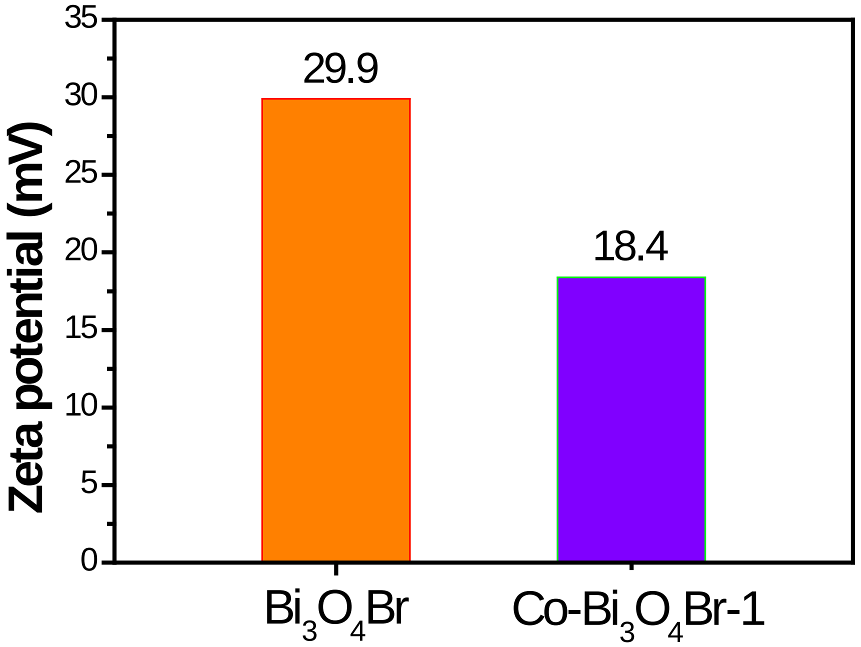

Supplementary Figure 5. Zeta potential measurements of Bi_3O_4Br and Co-Bi_3O_4Br-1 materials.

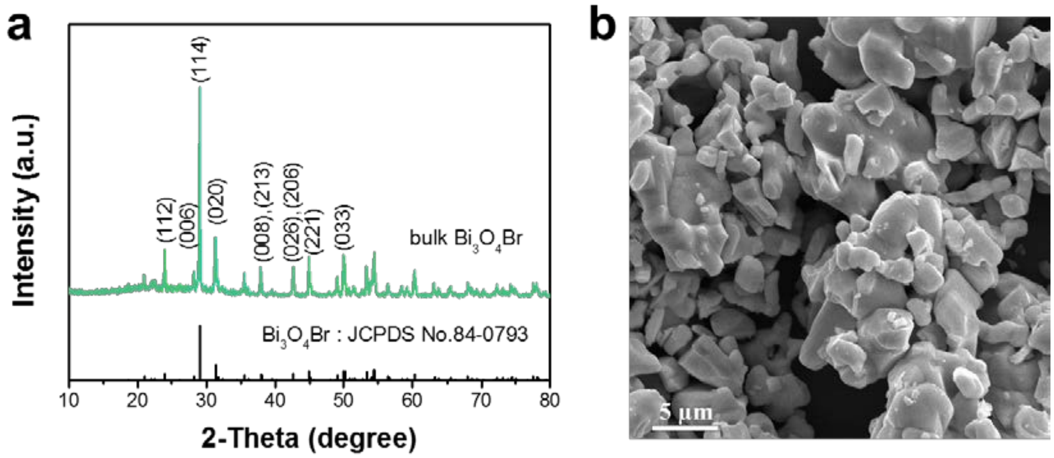

Supplementary Figure 6. Characterization of bulk Bi_3O_4Br. (a) XRD pattern and (b) SEM image of bulk Bi_3O_4Br.

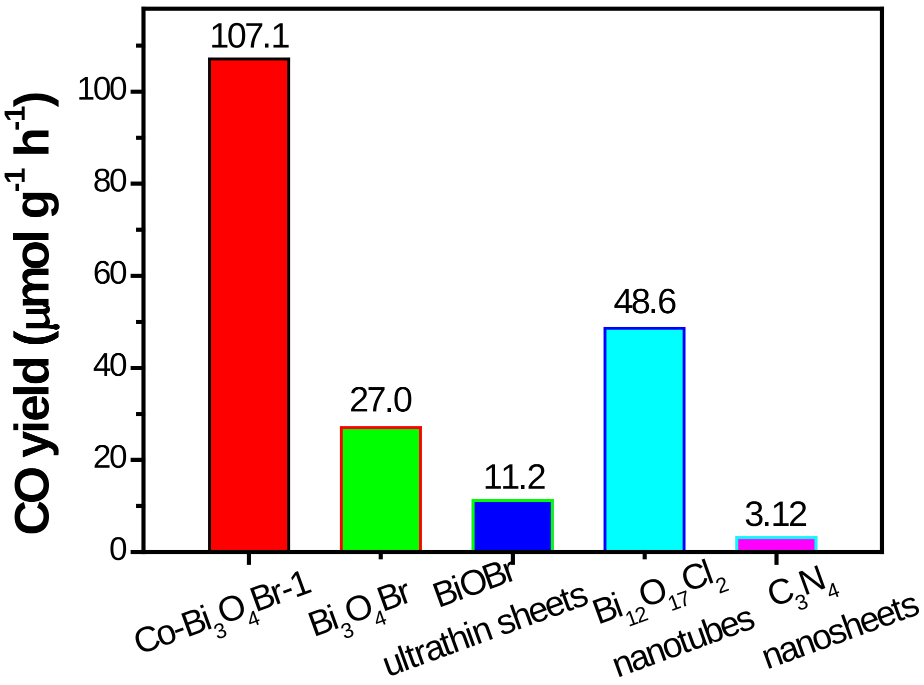

Supplementary Figure 7. Performance comparison of several ultrathin materials under the same testing conditions.

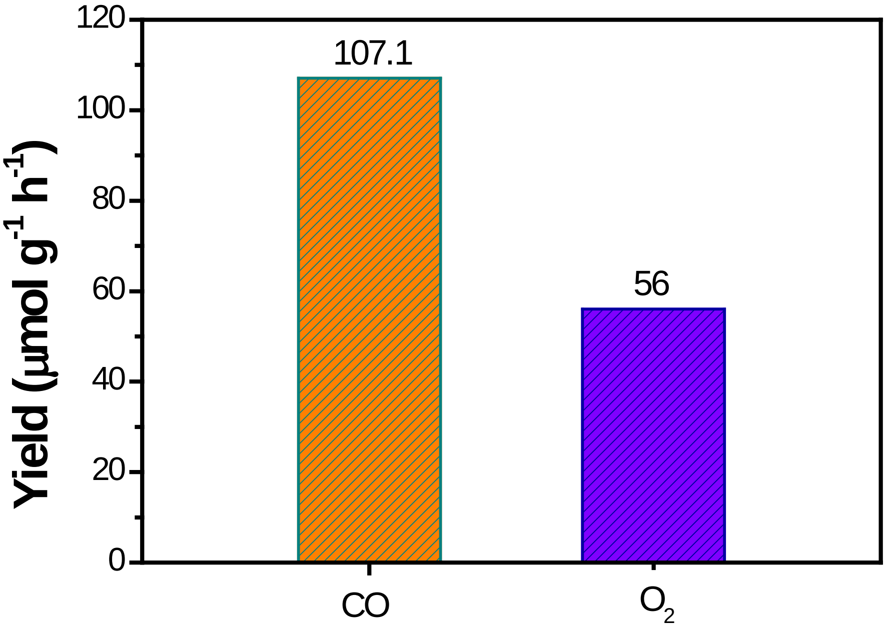

Supplementary Figure 8. Photocatalytic O_2 yield for the Co-Bi_3O_4Br-1 materials.

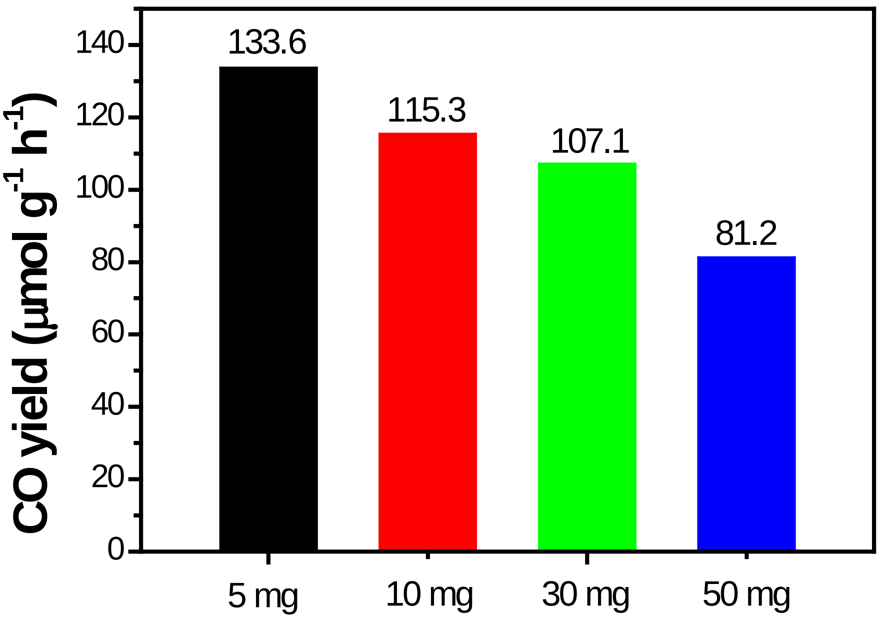

Supplementary Figure 9. Photoreduction of CO_2 into CO over Co-Bi_3O_4Br-1 with different usage amount of catalyst.

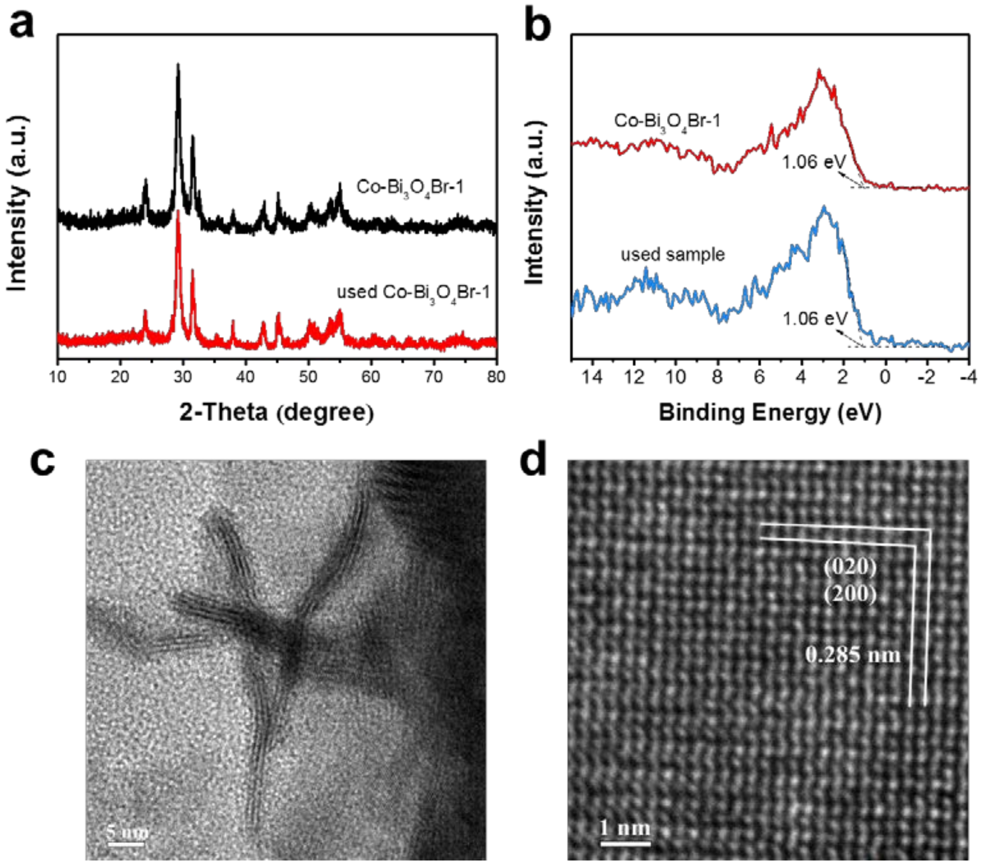

Supplementary Figure 10. Characterizations of Co-Bi_3O_4Br-1 after photocatalytic reaction. (a) XRD, (b) XPS valence band spectra and (c, d) TEM images.

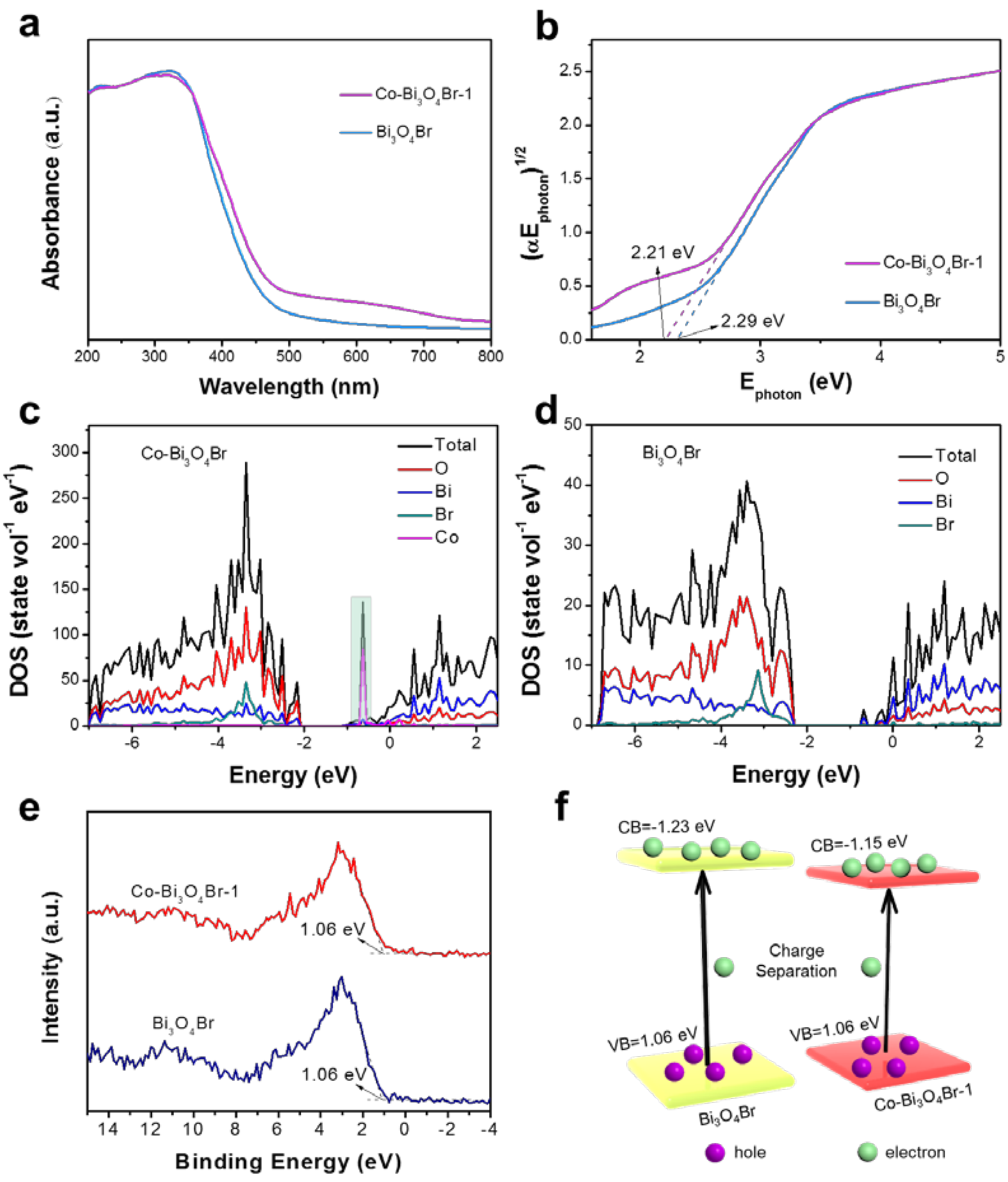

Supplementary Figure 11. Energy band structures. (a) UV/Vis diffuse reflection spectra and (b) Tauc plot of Bi_3O_4Br and Co-Bi_3O_4Br-1, calculated density of states of (c) Co-Bi_3O_4Br and (d) Bi_3O_4Br, (e) XPS valence band spectra of the Bi_3O_4Br and Co-Bi_3O_4Br-1, (f) schematic band structure obtained according to the results in (b) and (e).

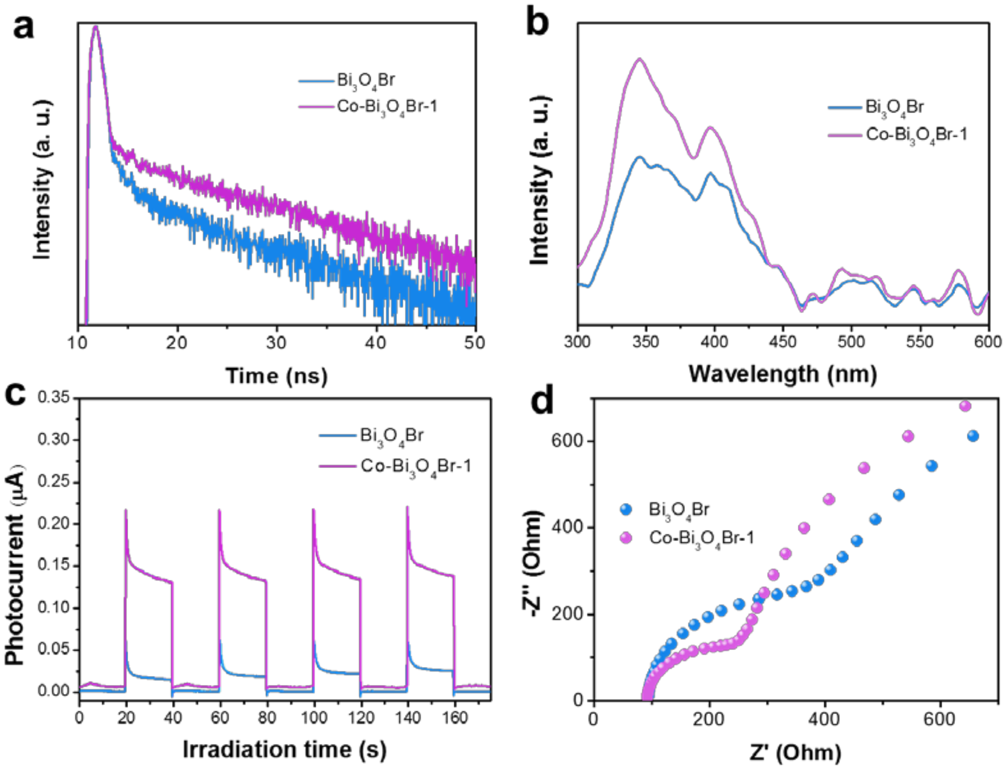

Supplementary Figure 12. Charge separation study. (a) Time-resolved transient PL decay, (b) surface photovoltage spectra, (c) transient photocurrent responses and (d) electrochemical impedance spectroscopy pure Bi_3O_4Br and Co-Bi_3O_4Br-1.

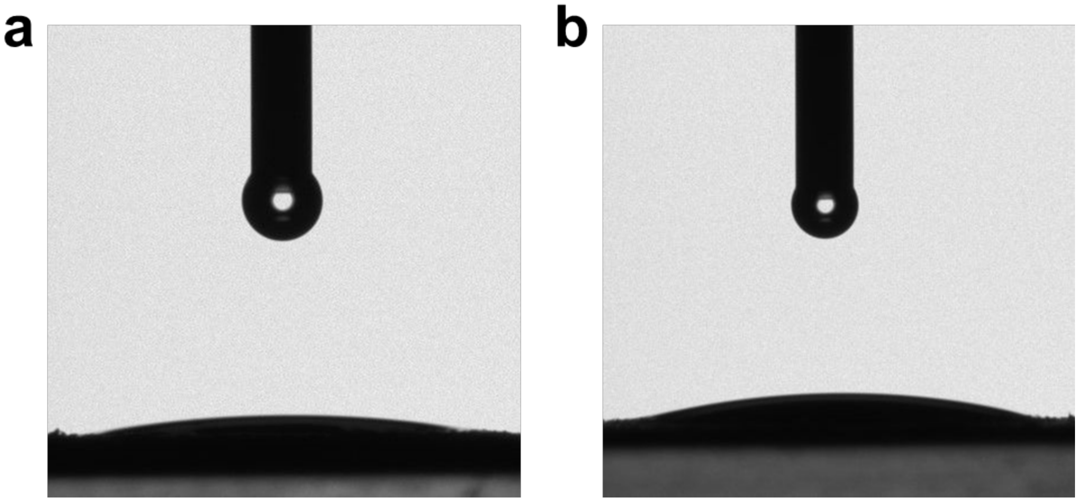

Supplementary Figure 13. Contact-angle. Static water contact-angle measurement of (a) Bi_3O_4Br and (b) Co-Bi_3O_4Br-1 materials.

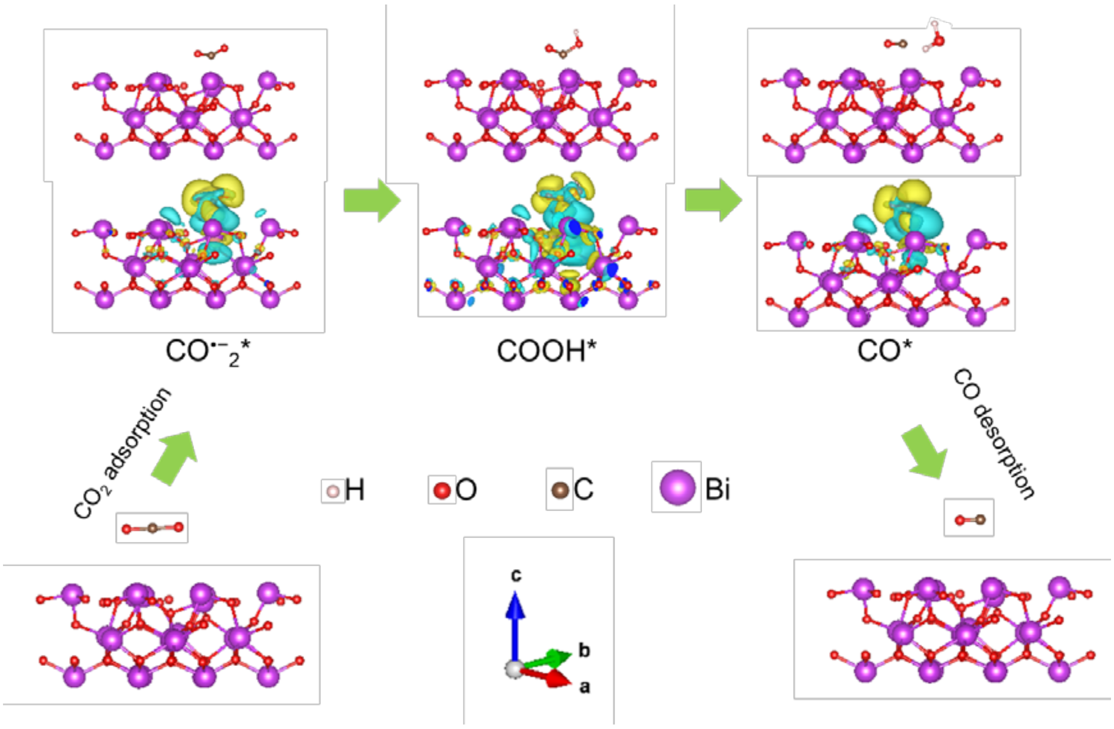

Supplementary Figure 14. Schematic representation of CO_2 photoreduction mechanism on the Bi_3O_4Br.

Supplementary Table 1. Comparison of the reaction conditions and photocatalytic activity with other catalysts for CO_2 reduction to CO.

**Photocatalysts**	**Light sources**	**Reaction conditions**	**Products**	**Photocatalytic efficiencies**	**Ref.**
Co-Bi_3O_4Br-1	Xe lamp	Liquid-solid, water	CO	107.1 μmol g^{-1} h^{-1}	This work
Oxygen-rich WO_3 layers	IR light	Liquid-solid, water	CO	2.8 μmol g^{-1} h^{-1}	1
Ultrathin ZnAl LDH	Xe lamp	Gas-solid, water	CO	7.6 μmol g^{-1} h^{-1}	2
Defect-rich $Bi_{12}O_{17}Cl_2$ nanotube	Xe lamp	Liquid-solid, water	CO	48.6 μmol g^{-1} h^{-1}	3
Oxygen-defect BiOBr atomic layers	Visible light	Liquid-solid, water	CO	87.4 μmol g^{-1} h^{-1}	4
BiOCl with oxygen vacancies	Xe lamp	Liquid-solid, water	CO	1.01 μmol g^{-1} h^{-1}	5
(001) facet exposed BiOBr	Xe lamp	Gas-solid, water	CO	4.45 μmol g^{-1} h^{-1}	6
(001) facet exposed BiOI	Xe lamp	Gas-solid, water	CO	5.18 μmol g^{-1} h^{-1}	7
$Bi_4O_5Br_2$	Visible light	Gas-solid, water	CO	2.73 μmol g^{-1} h^{-1}	8
Bi_4O_5BrI	Visible light	Gas-solid, water	CO	22.85 μmol g^{-1} h^{-1}	9
One-unit-cell $ZnIn_2S_4$	Xe lamp, AM 1.5G	Liquid-solid, water	CO	33.2 μmol g^{-1} h^{-1}	10
Partially oxidized SnS_2 atomic layers	Visible light	Gas-solid, water	CO	12.28 μmol g^{-1} h^{-1}	11
□ α-Fe_2O_3/g-C_3N_4	Xe lamp	Gas-solid, water	CO	27.2 μmol g^{-1} h^{-1}	12
UiO-66/C_3N_4	Visible	MeCN/TEOA = 4:1	CO	9.9 μmol g_{CN}^{-1} h^{-1}	13

	light				
BIF-20@g-C_3N_4	Visible light	MeCN/TEOA = 4:1	CO	53.869 μmol g^{-1} h^{-1}	14
Co tuned Au nanoclusters	Visible light	Liquid-solid, water/ TEOA	CO	3.45 μmol g^{-1} h^{-1}	15
Ni doped CdS quantum dots	Visible light	Liquid-solid, water/ TEOA	CO	~9.5 μmol g^{-1} h^{-1}	16

Supplementary References

1. Liang, L. *et al.* Infrared Light-Driven CO_2 Overall Splitting at Room Temperature. *Joule* **2**, 1-13 (2018).

2. Zhao, Y. F. *et al.* Defect-Rich Ultrathin ZnAl-Layered Double Hydroxide Nanosheets for Efficient Photoreduction of CO_2 to CO with Water. *Adv. Mater.* **27**, 7824-7831 (2015).

3. Di, J. *et al.* Defect-Rich $Bi_{12}O_{17}Cl_2$ Nanotubes Self-Accelerating Charge Separation for Boosting Photocatalytic CO_2 Reduction. *Angew. Chem. Int. Ed.* **57**, 14847-14851 (2018).

4. Wu, J. *et al.* Efficient Visible-Light-Driven CO_2 Reduction Mediated by Defect-Engineered BiOBr Atomic Layers. *Angew. Chem. Int. Ed.* **57**, 8719-8723 (2018).

5. Zhang, L. *et al.* Photoreduction of CO_2 on BiOCl nanoplates with the assistance of photoinduced oxygen vacancies. *Nano Res.* **8**, 821-831 (2015).

6. Wu, D., Ye, L. Q., Yip H. Y. & Wong, P. K. Organic-free synthesis of {001} facet dominated BiOBr nanosheets for selective photoreduction of CO_2 to CO. *Catal. Sci. Technol.* **7**, 265-271 (2017).

7. Ye, L. Q. *et al.* Facet-dependent photocatalytic reduction of CO_2 on BiOI

nanosheets. *Chem. Eng. J.* **291**, 39-46 (2016).

8. Ye, L. Q. *et al.* Thickness-ultrathin and bismuth-rich strategies for BiOBr to enhance photoreduction of CO_2 into solar fuels. *Appl. Catal. B* **187**, 281-290 (2016).

9. Bai, Y. *et al.* Synthesis of hierarchical bismuth-rich $Bi_4O_5Br_xI_{2-x}$ solid solutions for enhanced photocatalytic activities of CO_2 conversion and Cr(VI) reduction under visible light. *Appl. Catal. B* **203**, 633-640 (2017).

10. Jiao, X. C. *et al.* Defect-Mediated Electron−Hole Separation in One-Unit-Cell $ZnIn_2S_4$ Layers for Boosted Solar-Driven CO_2 Reduction. *J. Am. Chem. Soc.* **139**, 7586-7594 (2017).

11. Jiao, X. C. *et al.* Partially Oxidized SnS_2 Atomic Layers Achieving Efficient Visible-Light-Driven CO_2 Reduction. *J. Am. Chem. Soc.* **139**, 18044-18051 (2017).

12. Jiang, Z. F. *et al.* A Hierarchical Z-Scheme α-Fe_2O_3/g-C_3N_4 Hybrid for Enhanced Photocatalytic CO_2 Reduction. *Adv. Mater.* **30**, 1706108 (2018).

13. Shi, L. *et al.* Electrostatic Self-Assembly of Nanosized Carbon Nitride Nanosheet onto a Zirconium Metal–Organic Framework for Enhanced Photocatalytic CO_2 Reduction. *Adv. Funct. Mater.* **25**, 5360-5367 (2015).

14. Xu, G. L. *et al.* Integrating the g-C_3N_4 Nanosheet with B-H Bonding Decorated Metal-Organic Framework for CO_2 Activation and Photoreduction. *ACS Nano* **12**, 5333-5340 (2018).

15. Cui, X. F. *et al.* Turning Au Nanoclusters Catalytically Active for Visible-Light-Driven CO_2 Reduction through Bridging Ligands. *J. Am. Chem. Soc.* **140**, 16514-16520 (2018).

16. Wang, J. *et al.* Enabling Visible-Light-Driven Selective CO_2 Reduction by Doping Quantum Dots: Trapping Electrons and Suppressing H_2 Evolution. *Angew. Chem. Int. Ed.* **57**, 16447-16451 (2018).
